# Analysis of Elongational Viscosity of Entangled Poly (Propylene Carbonate) Melts by Primitive Chain Network Simulations

**DOI:** 10.3390/polym14040741

**Published:** 2022-02-14

**Authors:** Yuichi Masubuchi, Lixin Yang, Takashi Uneyama, Yuya Doi

**Affiliations:** Department of Materials Physics, Nagoya University, Nagoya 4648603, Japan; yang.lixin@g.mbox.nagoya-u.ac.jp (L.Y.); uneyama@mp.pse.nagoya-u.ac.jp (T.U.); ydoi@mp.pse.nagoya-u.ac.jp (Y.D.)

**Keywords:** viscoelasticity, entangled polymers, molecular simulations, rheology, coarse-graining

## Abstract

It has been established that the elongational rheology of polymers depends on their chemistry. However, the analysis of experimental data has been reported for only a few polymers. In this study, we analyzed the elongational viscosity of poly (propylene carbonate) (PPC) melts in terms of monomeric friction via primitive chain network simulations. By incorporating a small polydispersity of materials, the linear viscoelastic response was semi-quantitatively reproduced. Owing to this agreement, we determined units of time and modulus to carry out elongational simulations. The simulation with constant monomeric friction overestimated elongational viscosity, whereas it nicely captured the experimental data if friction decreased with increasing segment orientation. To see the effect of chemistry, we also conducted the simulation for a polystyrene (PS) melt, which has a similar entanglement number per chain and a polydispersity index. The results imply that PPC and PS behave similarly in terms of the reduction of friction under fast deformations.

## 1. Introduction

The nonlinear elongational rheology of polymers is out of the universality that has been established for the linear viscoelasticity and the nonlinear shear rheology [1]. According to the tube theory [2], the elongational viscosity of entangled polymers decreases with increasing strain rate when the elongation rate is located between the slowest relaxation rate and the Rouse relaxation rate reflecting the segment orientation. The viscosity shows an upturn at the strain rate comparable to the Rouse relaxation rate due to chain stretch. At the higher strain rates, the viscosity increases with increasing elongation rate, and it approaches a steady value that is determined by the maximum stretch ratio [3]. This behavior has been reported for polystyrene solutions [4,5,6,7] and a poly (n-butyl acrylate) (PnBA) melt [8]. However, Bach et al. [9] and Nielsen et al. [10] reported that the steady-state elongational viscosity of polystyrene (PS) melts monotonically decreases with increasing strain rate, even at elongation rates higher than the Rouse relaxation rate. Wingstrand et al. [11] reported that poly(methyl methacrylate) (PMMA) melts exhibit similar behavior with PS. Huang et al. [6,7] demonstrated that the elongational behavior of PS solutions is dependent on the PS concentration. These results indicate that the elongational behavior of polymeric liquids is chemistry-dependent and not universal, unlike the shear rheology.

For the elongational thinning, a few different molecular mechanisms have been proposed [1,12,13]. Among them, we focus on the change of monomeric friction [1] in this study. Ianniruberto et al. [14] performed atomistic molecular simulations of a melt of styrene oligomers to report a decrease of segmental friction under fast flows due to segment orientation. Following this work, Yaoita et al. [15] analyzed stress relaxation after fast elongation of a PS melt and a PS solution to extract monomeric friction. They reproduced the elongational behavior by incorporating the friction change into multi-chain slip-link simulations. The simulation has been successfully applied to various PS melts and solutions [16], including bidisperse [17] and branch systems [18,19] to demonstrate the consistency of the friction change for different systems. Similar studies have been conducted for molecular constitutive equations [1,20,21,22,23,24].

As summarized in a recent review [1], the change of friction is not universal, but is dependent on the chemistry of the polymers. The melt data for PS, PMMA, and PI suggest a similar friction change for these polymers, whereas the data of a PnBA melt imply no friction change. Matsumiya et al. [25] examined unentangled PS and poly(t-butyl styrene) (PtBS) melts to report that they exhibit the reduction of friction in different manners. Morelly et al. [26] reported similar data for a PtBS melt and PMMA melts. The change of friction has also been discussed for coarse-grained, bead-spring chains [1,27,28,29], although it is still controversial.

However, the effect of chemistry on the friction change has not yet been clarified. Masubuchi et al. [16] speculated that for the case of PnBA, the flexible side group prevents orientation among the main chains. According to this argument, the friction change of PtBS would be suppressed by the methyl group added to the benzyl group of PS. The results by Matsumiya et al. [25] are consistent with this thought. However, such a discussion is difficult for PMMA [26]. An ideal benchmark would be polyethylene, for which elongational data are available for some systems with small polydispersity indices [30]. However, crystallization and small rheological activation energy do not allow us to measure the behavior under fast and high stretch. As such, a possible option would be carbonate polymers that have no bulky side group and no crystal formation.

In this study, we analyzed elongational data of a few poly (propylene carbonate) (PPC) melts to discuss the friction change. This carbonate polymer does not have bulky side groups, and it does not exhibit crystallization under the usual experimental conditions. Since no data for monodisperse PPC are available, we employed the dataset for polydisperse PPC melts [31,32], for which linear and nonlinear rheological properties have been reported. Although the polydispersity index is 1.3, which is not too large, we took account of its effects by using the multi-chain slip-link model. After confirming consistency for linear viscoelasticity, we calculated the nonlinear viscoelasticity under fast elongation to evaluate the friction change. Details are shown below.

## 2. Model and Simulations

We employed the multi-chain slip-link simulation based on the primitive chain network (PCN) model [33,34,35,36,37], although other molecular models could also be used as long as elongational rheology can be calculated for polydispersed systems with practical computation costs. Since the simulation code and the method are the same as those employed in earlier studies for elongational rheology [15,16,17,18,19,38,39,40,41,42], readers familiar with them can skip this section.

In the model, we replace entangled polymer melts with slip-link networks that consist of network strands, nodes, and dangling ends. Each polymer corresponds to a path connecting two dangling ends through some strands and connected nodes. At each network node, one slip-link is placed to bundle two polymer chains according to the binary assumption of entanglement. We describe the dynamics by the 3-dimensional motion of the slip-links, chain sliding through the slip-links, and the creation and destruction of the slip-links at the chain ends. The slip-link position obeys a Langevin-type equation of motion. The force balance is considered among drag force, tension acting on the diverging strands, osmotic force suppressing density fluctuations, and thermal random force. The chain sliding through each slip-link is calculated by a change rate equation of the number of Kuhn segments between consecutive strands. In the calculation, the force balance incorporated for the slip-link motion is considered along the chain. When a dangling end slides off from the connecting slip-link due to chain sliding, the slip-link is removed, and the bundled chain is released. Vice versa, when a dangling end protrudes from the connecting node, a new network node is created by hooking one of the surrounding strands.

We choose units of length, energy, and time as the average strand length a, thermal energy $k_{B}T$, and the diffusion time of a single network node $\tau_{0}=2n_{0}\zeta_{0}a^{2}/6k_{B}T$. Here, $\zeta_{0}$ is the friction of a single Kuhn segment under equilibrium, and $n_{0}$ is the average number of Kuhn segments on network strands. The factor of two in $\tau_{0}$ means that $2n_{0}$ Kuhn segments are accommodated on a single network node on average.

We note that these parameters could be determined from atomistic and coarse-grained molecular simulations, as attempted earlier [43,44,45,46,47,48]. However, we did not employ such a bottom-up approach due to practical difficulties for atomistic simulations of PPC. Instead, we determined the parameters empirically via fitting of linear viscoelasticity. Namely, we optimized units of modulus $G_{0}$ and molecular weight $M_{0}$, instead of a, following the common strategy for entanglement-based modeling [37,49,50,51]. The determined parameter values are $G_{0}=1.9$ (MPa), $M_{0}=3.3$ (kg/mol), and $\tau_{0}=7.8\times 10^{-2}$ (s) at T=70 °C. Note that $G_{0}$ and $M_{0}$ are similar to, but different from, the plateau modulus $G_{N}$ and the entanglement molecular weight $M_{e}$ as reported earlier [34,36,51]. For the case of PPC, $G_{N}$ and $M_{e}$ determined from linear viscoelasticity are reported as 670 (kPa) (at 373 K) and 5.9 (kg/mol). The difference is due to fluctuations imposed on network nodes in the PCN model [34,36,51,52,53]. As assumed for other entanglement-based models [49,50], these parameter values are not dependent on the molecular weight [34,36,54], its distribution [17,55], and the long-chain branching [18,19,35,56,57,58,59]. $G_{0}$ and $\tau_{0}$ depend on temperature by their construction. ($G_{0}$ is thermal energy per unit volume.) The parameters are insensitive to flow and deformation [15,35,38,57,60,61,62,63,64].

To consider the molecular weight distribution, we set up simulation systems as mixtures of polymers with different molecular weights, as shown in Table 1. Due to practical limitations, we had four components for each case, and the molecular weight and the chain number fraction of each component were determined assuming a log-normal distribution. For the resultant set of molecules, the weight average number of entanglements, $Z_{w}=M_{w}/M_{0}$, and the polydispersity index, $Z_{w}/Z_{n}$, are consistent with the target experiment [31,32]. For comparison, we also performed simulations for a monodisperse system.

Following earlier studies [15,16,17,19,38,59], we incorporated finite chain extensibility (FENE) and the change of monomeric friction according to the orientation and stretch of polymers. FENE is considered via the FENE-P approximation [65], in which the spring constant of each component, $f_{\mathrm{FENE},\;i}$, is calculated from the averaged stretch of strands by the following equation.

(1) $$f_{\mathrm{FENE},\;i}=\frac{1}{1-\langle {\tilde{\lambda }}^{2}\rangle_{i}}\;$$

Here, $\tilde{\lambda }$ is the normalized stretch of strands concerning the maximum stretch $\lambda_{\max}$, and the brackets, ${\langle \cdots \rangle }_{i}$, mean the ensemble average for each component. $\lambda_{\max}$ is determined by the number of Kuhn segments on a single strand, $n_{K}$, as $\lambda_{\max}=\sqrt{n_{K}}$. From the molecular weight of the single Kuhn segment for PPC $m_{K}=147$ g/mol estimated earlier [32], $n_{K}=M_{0}/m_{K}\sim 22.7$ and $\lambda_{\max}=4.8$ in our model. We also examined a PS melt, for which $\lambda_{\max}=3.9$ [38].

As demonstrated later, simulations only equipped with FENE cannot reproduce the elongational behavior. Thus, we examined the friction change proposed by Ianniruberto et al. [14,22].

(2) $$\frac{\zeta \left(S\right)}{\zeta_{0}}=\left\{\begin{array}{cc} 1 & \mathrm{for}\;S<S_{c}\\ {\left(S/S_{c}\right)}^{-\alpha } & \mathrm{for}\;S\geq S_{c} \end{array}\right.\;$$

Here, S is strand orientation defined as $S=\langle u_{x}^{2}\rangle -\langle u_{y}^{2}\rangle$. $u=\left(u_{x},\;u_{y},u_{z}\right)$ is the strand orientation vector, and x and y are the elongational and perpendicular directions, respectively. $S_{c}$ and α are phenomenological parameters, and we chose them to be $S_{c}=0.1$ and α=1.25. We note that other functional forms of friction have been proposed [15,21]. Although we restrict our discussion to Equation (2), one may employ these friction models with tuning the model parameters.

**Figure 1:**
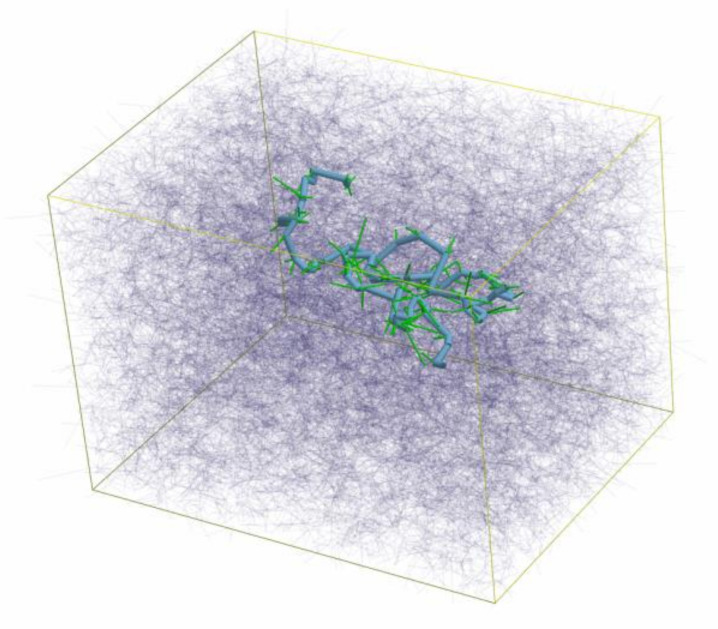
Typical snapshot of a chain with Z=88 involved in Zw48 under elongation with the elongation rate of $\dot{\varepsilon }\tau_{0}=4.4\times 10^{-5}$, taken at the elongational strain of 1.76. Thin black lines are the other chains, and thick green lines are segments entangled to the test chain. The yellow frame shows the simulation box for periodic boundary conditions.

We performed simulations to obtain linear and nonlinear viscoelasticity. The linear relaxation modulus, $G\left(t\right)$, was obtained from stress fluctuations according to the Green–Kubo formula under equilibrium. We employed periodic boundary conditions for a simulation box with the box dimension of 12^3^, which is sufficiently large to accommodate the examined polymers. The equilibrium simulations were performed for a sufficiently long time, and the duration was at least 10 times longer than the longest relaxation time for each system. For statistics, $G\left(t\right)$ was averaged over 32 independent simulation runs. The obtained $G\left(t\right)$ was converted to storage and loss moduli, $G^{\prime }\left(\omega \right)$ and $G^{\prime \prime }\left(\omega \right)$, via the REPTATE software [66]. For uniaxial deformation, following the previous studies [15,38], we equilibrated the system filled in a flat simulation box and stretched it. The dimension of the initial flat box was 4 × 45 × 45, and that at the final elongated state was 506 × 4 × 4. This deformation attains the Hencky strain of 4.8. We acquired the uniaxial viscosity growth function, $\eta_{E}{}^{+}\left(t\right)$, by averaging the stress growth in 8 independent simulation runs for each condition. Figure 1 exhibits a typical snapshot of the longest chain with Z=88 during the elongation, with the elongation rate of $\dot{\varepsilon }\tau_{0}=4.4\times 10^{-5}$, and the strain of 1.76.

## 3. Results and Discussion

**Figure 2:**
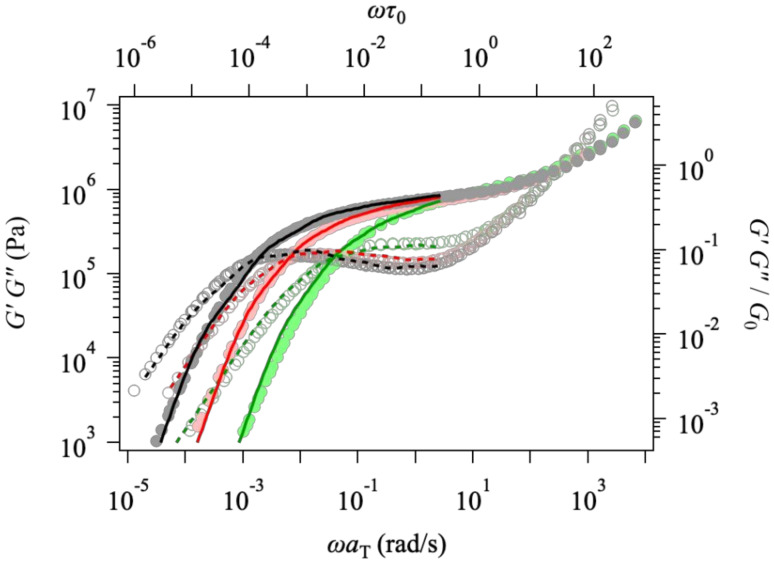
Linear viscoelasticity at T=70 °C from experiments [31] (shown by symbols and plotted against the bottom and left axes) and simulations (indicated by curves and plotted against the top and right axes) for Zw47 (PPC158k), Zw34 (PPC111k), and Zw21 (PPC69k) from left to right.

Figure 2 shows the linear viscoelastic response $G^{\prime }\left(\omega \right)$ and $G^{\prime \prime }\left(\omega \right)$. The simulation results (displayed by colored curves) semi-quantitatively capture the experiment (indicated by circles) for all the examined samples. As discussed later, the polydispersity is not significant, yet not negligible, for the viscoelastic spectrum, and the agreement is due to the multi-chain nature [17,55]. According to this comparison, the basic parameters $G_{0}$ and $\tau_{0}$ were determined as mentioned in the previous section and used for simulations discussed hereafter.

**Figure 3:**
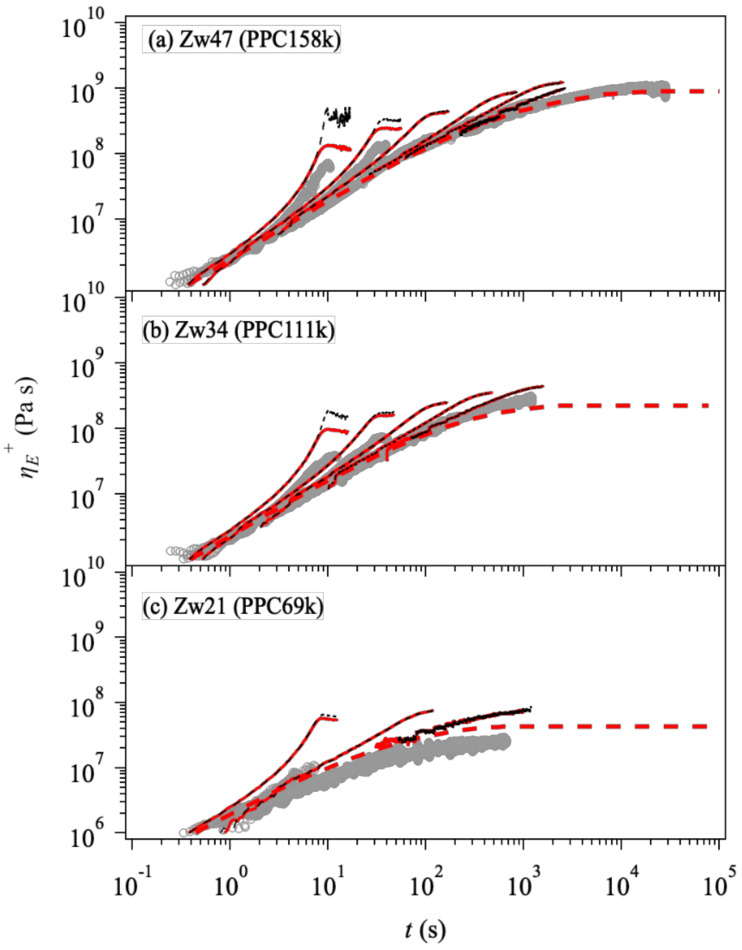
Viscosity growth curves under uniaxial elongation at T=70 °C for Zw47 (PPC158k, panel (**a**)), Zw34 (PPC111k, panel (**b**)), and Zw21 (PPC69k, panel (**c**)) from top to bottom. Experimental data [32] are shown by circles. Simulation results with and without the change of friction (according to Euqation (2)) are indicated by red solid and black broken curves. Red broken curves show the linear viscoelastic envelopes. The strain rates are $2.8\times 10^{-1},\;8.5\times 10^{-2},\;2.8\times 10^{-2},\;5.6\times 10^{-3},\;1.8\times 10^{-3},\;5.6\times 10^{-4},\;\mathrm{and}\;1.8\times 10^{-4}$ s^−1^ for Zw47 (PPC158k), $3\times 10^{-3},\;1\times 10^{-2},\;3\times 10^{-2},\;1\times 10^{-1},\;\mathrm{and}\;3\times 10^{-1}$ s^−1^ for Zw34 (PPC111k), and $4\times 10^{-3},\;6.1\times 10^{-3},\;4\times 10^{-2},\;6.1\times 10^{-2},\;4\times 10^{-1},\;\mathrm{and}\;6.1\times 10^{-1}\;$ s^−1^ for Zw21 (PPC69k), respectively.

Figure 3 exhibits elongational viscosity growth curves, $\eta_{E}{}^{+}\left(t\right)$. The simulation results are not dependent on the friction change when the stretch rate is relatively low, as shown by the overlapped red solid and black broken curves. As the stretch rate increases, the simulation with the constant monomeric friction (black, broken curve) vastly overestimates the data, implying that the friction is reduced under these conditions. Indeed, the simulation with the friction change (solid red curve) better captures the experiment under fast stretch. See the leftmost curves in all the panels. To be fair, we note that the simulation overestimates the data for PPC69k (panel c), even for the linear viscoelastic envelope shown by the red broken curve. As we confirmed the consistency between the linear viscoelastic envelope and $\eta_{E}{}^{+}\left(t\right)$ for the simulation, this discrepancy is due to experimental difficulties for the elongational experiment for this low viscosity material. Note also that the difference is enhanced in the double-log plot.

**Figure 4:**
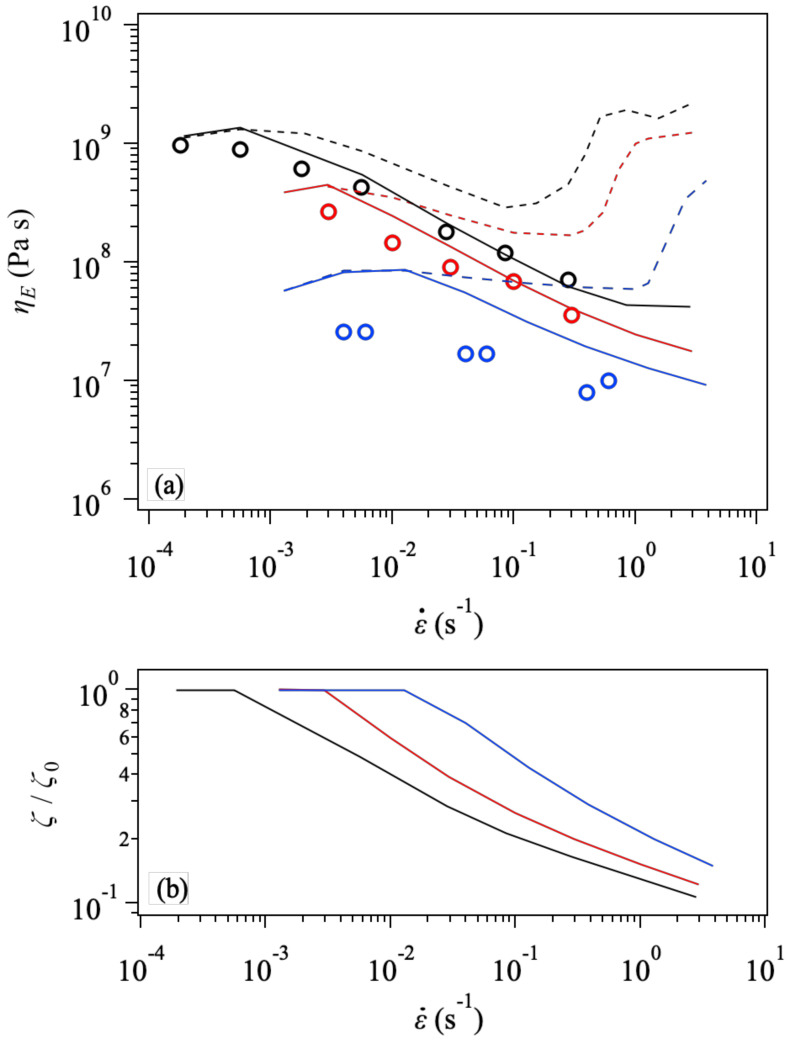
Steady-state elongational viscosity (**a**) and the friction (**b**) at T=70 °C as a function of the strain rate for Zw47 (PPC158k, black), Zw34 (PPC111k, red), and Zw21 (PPC69k, blue). Experimental data [32] are shown by unfilled circles. In the top panel (**a**), simulation results with and without the friction change (according to Equation (2)) are drawn by solid and broken curves, respectively.

The effect of friction change can be clearly seen in Figure 4, in which the steady-state viscosity and friction as functions of the strain rate are shown. In the top panel, $\eta_{E}$ is close to $3\eta_{0}$ at small $\dot{\varepsilon }$. (Here, $\eta_{0}$ is the zero-shear viscosity determined from the linear viscoelastic response.) As $\dot{\varepsilon }$ increases, $\eta_{E}$ decreases due to the orientation [1,2]. Without the friction change, as mentioned in the introduction, $\eta_{E}$ shows an upturn reflecting chain stretch. According to the theory [1,3], the critical $\dot{\varepsilon }$ for the upturn is close to the Rouse relaxation ratio. However, because of the polydispersity of our system, we cannot define a single Rouse time. In this respect, the critical stretch ratio for the upturn is hardly predicted without the simulation. Nevertheless, such an upturn is not observed in the experimental data (see circles), which exhibit a monotonic decrease with increasing $\dot{\varepsilon }$. The simulation captures this monotonic trend with the friction change. See the solid curves in the panel (a), which deviate from the constant friction case shown by broken curves around the strain rate close to the reciprocal longest relaxation time. The slope of $\eta_{E}$ against $\dot{\varepsilon }$ before the upturn is more rapid than the case without the friction change. Although the data for PPC69k (blue symbols) are not quantitatively reproduced as mentioned in Figure 3, the simulation with the friction change better predicts the data. The panel (b) exhibits that the friction is reduced to 10% concerning the equilibrium value.

**Figure 5:**
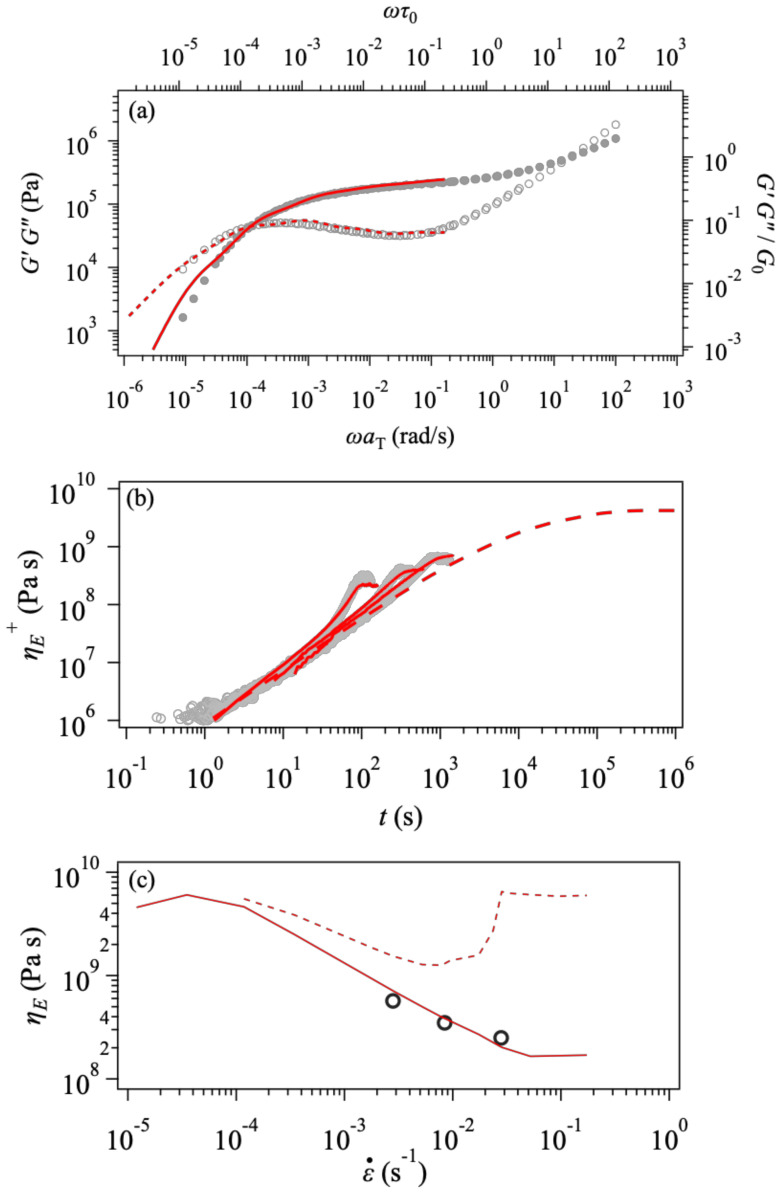
Simulation results for a polystyrene melt with $M_{w}=520\;k$ and $M_{w}/M_{n}=$ 1.3 for linear viscoelasticity (**a**), elongational growth curve (**b**), and steady-state elongational viscosity (**c**) at T=130 °C. Circles and curves are experimental and simulation results, respectively. Solid and broken curves in the panel (**b**) exhibit the simulation results with the friction change and the linear viscoelastic envelope. The strain rates are $2.8\times 10^{-4}$, $8.4\times 10^{-4}$, and $2.8\times 10^{-3}$ s^−1^, from left to right. In the panel (**c**), solid and broken curves correspond to the simulation with and without the friction change.

To see the effect of chemistry, we performed additional simulations for a PS melt with $M_{w}=520\;k$ and $M_{w}/M_{n}=$ 1.3. For this PS melt, the experimental data were taken by the same operator and apparatus [67] as those for the PPC examined above. Since the values of $Z_{w}$ and $Z_{w}/Z_{n}$ are close to those for PPC158 k, we used Zw47 with the parameters chosen as $\left(M_{0},\;\lambda_{\max}\right)\;=\;$(11 kg/mol, 3.9) according to earlier studies [15,16,17,18,19]. The top panel of Figure 5 shows the linear viscoelasticity at T=130 °C. As expected, the simulation reasonably reproduces the data, yet $G^{\prime }$ in the terminal regime is slightly overestimated due to the discrepancy in higher moments of molecular weight distribution than those indicated by $M_{n}$ and $M_{w}$. Nevertheless, the other parameters are determined from this fitting as $\left(G_{0},\tau_{0}\right)\;=$ (0.55 MPa, 1.3 s). We performed elongational simulations using the determined parameter set according to friction described by Equation 2. Note that the parameter values are common with PPC ($S_{c}=0.1$ and α=1.25). The predicted elongational viscosity growth curves are shown in the mid panel, exhibiting reasonable agreement with the experiment. The bottom panel shows the steady-state viscosity, $\eta_{E}\left(\dot{\varepsilon }\right)$. These elongational results demonstrate that the simulation can reproduce the data for PPC and PS by incorporating the same friction model. To be fair, we note that the simulation slightly overestimates the data if the comparison is made for the low strain rates due to the deviation in the linear viscoelasticity. We also note that the magnitude of friction change for this specific PS melt is larger than that reported earlier for the other PS systems [15,16,17,18,19]. Nevertheless, the data for PPC and PS imply that these two polymers behave in a similar manner under elongation, despite the difference in their chemistry.

**Figure 6:**
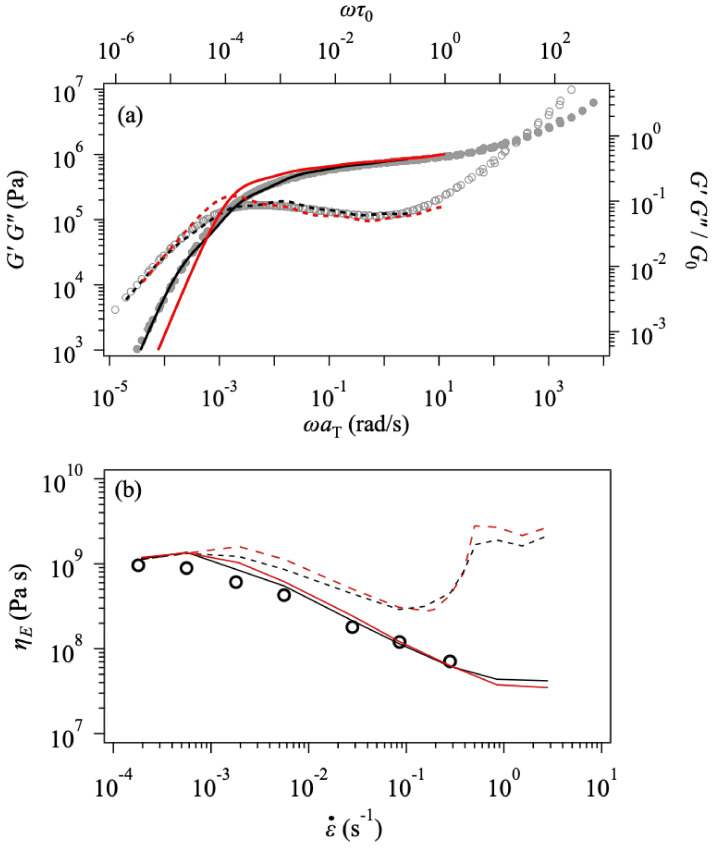
Comparison between Zw47 (the polydispersity index of 1.3, black curves) and Z47 (monodisperse, red curves) for linear viscoelasticity (**a**) and steady-state elongational viscosity plotted against strain rate (**b**). The experimental data for PPC158 k are also shown by symbols.

Let us turn our attention to the effect of polydispersity. Takeda et al. [17] have performed PCN simulations for bidisperse polystyrene melts. They reported that friction remains constant even under fast stretch when the short-chain fraction reduces the stretch-orientation order parameter. This result implies that the friction change depends on polydispersity. To see if the polydispersity in Zw47 changes friction, we performed a comparison between polydisperse (Zw47) and monodisperse (Z47) cases, as shown in Figure 6. Even though the polydispersity index is 1.3, which is not large, the effect is seen for linear and nonlinear viscoelasticity. As established earlier, widely distributed relaxation modes in the polydisperse system loosen the terminal behavior in $G^{\prime }$ and $G^{\prime \prime }$, and the modulus at the crossover between $G^{\prime }$ and $G^{\prime \prime }$ is reduced, reflecting the zero-shear compliance. Meanwhile, the zero-shear viscosity is not affected by the polydispersity, as $G^{\prime \prime }$ curves overlap entirely with each other. The plateau modulus is also insensitive to polydispersity. Concerning the elongational viscosity shown in the bottom panel, without the friction change (broken curves), the viscosity decreases with increasing strain rate with a power-law manner with the exponent of ca. −1/2. This slope becomes slightly mild for the case with polydispersity. The viscosity upturn is observed at $\dot{\varepsilon }\sim 0.2\;s$^−1^ for both cases, followed by the steady-state at the large $\dot{\varepsilon }$ limit. Although the value of $\lambda_{\max}$ is common, the viscosity for the polydisperse case is slightly suppressed by the contribution of low molecular weight components. With the friction change (solid curves), the decrease of viscosity with increasing $\dot{\varepsilon }$ is similar for both cases. According to this result, we conclude that the effect of polydispersity on the friction change is not significant for this specific case due to the small polydispersity index.

## 4. Conclusions

We performed primitive chain network simulations for a few PPC melts to analyze elongational behavior in terms of the friction change. The linear viscoelasticity was semi-quantitatively reproduced if the polydispersity was considered. Owing to this agreement, we determined the model parameters to conduct elongational simulations. The simulation overestimated the elongational viscosity if monomeric friction was kept constant. Meanwhile, the viscosity was nicely captured if friction changed according to the segment orientation, as proposed by Ianniruberto et al. [14,22]. The effect of the polydispersity on the elongational behavior was negligible in the examined case with the polydispersity index of 1.3. To see the effect of chemistry, we also conducted simulations for a PS melt, which has a similar entanglement number per chain and molecular weight distribution to one of the examined PPC melts. The simulation results for this PS were in good agreement with the data if the same friction model with PPC was applied.

The result implies that the elongational behavior of PPC is similar to that of PS in terms of the friction change. If this conclusion can be applied to the nonlinear rheology of PPC in general, the modeling of PPC rheology is straightforward. However, to be fair, we note that the analysis has been done only for the limited experimental data. Further investigation is necessary for other samples and other nonlinear flows, such as fast shear. We also note that the conclusion may change if the analysis is made according to the other molecular mechanisms proposed for elongational rheology [13,68].

## Figures and Tables

**Table 1:** Examined systems.

Code	Z	$\varphi_{N}{\;}^{\#}$	$Z_{w}$	$Z_{w}/Z_{n}$	${M_{w}}^{+}\;(\mathrm{kg}/\mathrm{mol})$	$M_{w}/{M_{w}}^{+}$
Zw47 (PPC158k *)	11	0.09	47.3	1.31	158	1.30
	22	0.41				
	44	0.41				
	88	0.09				
Zw34 (PPC111k *)	8	0.09	33.7	1.30	111	1.30
	16	0.41				
	32	0.41				
	62	0.09				
Zw21 (PPC69k *)	4	0.1	20.6	1.41	68.8	1.43
	8	0.4				
	18	0.4				
	38	0.1				
Z47	47	1	47	1	-	-

* Sample code for examined PPC melts [31,32]. ^#^ Number fraction of the chains. ^+^ Values reported according to GPC-MALS.

## Data Availability

The data presented in this study are available on request from the corresponding author.
